# Tau–Mitochondria Interactions in Neurodegeneration: Mechanisms and Therapeutic Potential

**DOI:** 10.1007/s10571-025-01634-1

**Published:** 2025-11-25

**Authors:** Yaisa Castillo-Casaña, Clorinda Arias, Roberto Coria

**Affiliations:** 1 Departamento de Bioquímica y Biología Estructural, Instituto de Fisiología Celular, Universidad Nacional Autónoma de México (UNAM), Cd Mex, 04510 México; 2 Departamento de Medicina Genómica y Toxicología Ambiental, Instituto de Investigaciones Biomédicas, Universidad Nacional Autónoma de México (UNAM), Cd Mex, 04510 México

**Keywords:** Tau protein, Mitochondrial dysfunctions, Mitochondrial bioenergetics, Mitochondrial transport, Mitochondrial dynamics, Mitophagy, Neurodegeneration, Tauopathies

## Abstract

Tau is a microtubule-associated protein encoded by the MAPT gene and is mainly expressed in neurons. Alternative splicing generates preferentially six isoforms differing in N-terminal inserts (0, 1, or 2N) and microtubule-binding repeats (3R or 4R). Isoform expression varies by cell type, developmental stage, and neuronal maturation. Structurally, 4R isoforms bind and stabilize microtubules more effectively than 3R isoforms, while 3R variants are more prone to oligomerization. Differences among isoforms also affect aggregation and post-translational modification patterns, yet their specific roles in tauopathies remain unclear. Beyond its role in microtubule stabilization, tau is increasingly recognized for its functions in other cellular compartments, particularly mitochondria, where it may contribute to mitochondrial dysfunction in neurodegenerative diseases. Its intrinsically disordered conformation and extensive post-translational modifications enable interactions with multiple mitochondrial components, linking tau biology to broader aspects of neuronal health and pathology. The main focus of this review is to analyze how tau protein interacts with mitochondria and disrupts their function. Literature evidence indicates that tau localizes to the outer mitochondrial membrane, intermembrane space, and matrix, where it interferes with key processes. These include disruption of electron transport chain activity, inhibition of ATP synthase, and reduced ATP production, ultimately compromising neuronal energy supply. In parallel, tau destabilizes microtubule-based trafficking, impairing axonal transport and mitochondrial distribution, while also disrupting fission and fusion dynamics that shape mitochondrial morphology. Quality control pathways are affected as well, with tau altering mitophagy and mitochondria-nucleus signaling. Moreover, tau dysregulates calcium buffering and increases reactive oxygen species production, thereby promoting synaptic dysfunction, oxidative stress, and mitochondrial damage. Collectively, these facts establish tau as a central mediator of mitochondrial impairment and neuronal vulnerability. Elucidating the mechanisms by which tau affects mitochondrial physiology underscores its importance as a therapeutic target, with strategies aimed at preserving mitochondrial integrity offering promising avenues to slow neurodegenerative progression. In the last section, we include examples of clinical applications currently in various phases of testing, some of which show promising potential for implementation.

## Introduction

Neurodegenerative diseases are a group of complex disorders characterized by abnormal protein aggregation in the central or peripheral nervous system, leading to progressive neuronal death and functional decline (Gadhave et al. 2024). The exact causes of many of them remain unclear, posing significant challenges for treatment development. Proteins such as α-synuclein, huntingtin, TDP-43, β-amyloid, and tau have been implicated in neurodegenerative diseases called proteinopathies (Riguet et al. 2021; Moda et al. 2023; Tsoi et al. 2023). Among them, tau is particularly significant due to its essential role in neuronal functions, and because its oligomerization is closely linked to neuronal death and cognitive decline (Silva and Haggarty 2020). The primarily biological function of tau is to provide dynamic stability to axonal microtubules (Iwata et al. 2019), and establish neuronal polarity (Mandell and Banker 1995; Kempf et al. 1996). However, hyperphosphorylated forms of tau has also been observed in the somatodendritic domain, correlating with synaptic loss and early neurodegeneration. Intriguingly, tau may also be present in nuclei and near ribosomes, which are rich in nucleic acid (Papasozomenos and Binder 1987) and in mitochondria (Tracy et al. 2022), indicating roles beyond cytoskeletal regulation. Mitochondrial dysfunction is recognized as an early feature of tauopathies (Palikaras et al. 2021), and this article explores the evidence for tau’s mitochondrial localization and its functional implications.

### Tau Structure

Tau was first characterized in 1975 as a microtubule-associated protein (MAP) essential for microtubule dynamics (Weingarten et al. 1975). It is predominantly expressed in neurons of the central and peripheral nervous systems and is encoded by the MAPT gene, located on chromosome 17. Of the 16 exons comprising the MAPT gene, exons 1, 4, 5, 7, 9, 11, 12, and 13 are constitutively expressed, defining the main structural domains of tau: the N-terminal end (exon 1), the proline-rich domain (exons 4, 5, 7, and part of 9), the microtubule-binding domain (part of exon 9, 11, and 12), and the C-terminal end (exon 13) (Corsi et al. 2022; Buchholz and Zempel 2024). Alternative splicing of exons 2, 3, 4a, 6, 8, and 10 gives rise to multiple isoforms. Inclusion of exons 2 and 3 inserts short amino acid sequences at the N-terminus, whereas exon 10 encodes the second repeat in the microtubule-binding domain (Ruiz-Gabarre et al. 2022; Buchholz and Zempel 2024). Consequently, six tau isoforms are expressed in the adult human brain, differing in N-terminal inserts (0, 1, or 2N) and microtubule-binding repeats (3R or 4R) (Buchholz and Zempel 2024). Tau splicing is strictly regulated and depends on the cell type, neuronal maturation, and developmental stage (Fiock et al. 2020; Buchholz and Zempel 2024). During fetal neurogenesis, only the 0N3R isoform is expressed, whereas the synthesis of other isoforms increases progressively throughout neuronal and individual development (Bachmann et al. 2021).

Although the precise biological significance of multiple isoforms remains unclear, their differential expression during development suggests distinct functions. N-terminal inserts appear to regulate both subcellular localization and interaction networks: 0N isoforms are mainly axonal and somatic, 1N isoforms localize to nuclei and dendrites, and 2N isoforms are broadly distributed (Bachmann et al. 2021). These localization patterns reflect distinct protein–protein interactions and cellular roles. Structural differences also modulate microtubule affinity: 4R isoforms bind microtubules more strongly, better suppress shortening events, and stabilize disassembly intermediates more efficiently than 3R isoforms (Best et al. 2019). In contrast, unmodified 3R isoforms are more prone to oligomerization (Shahpasand-Kroner et al. 2022), and the presence of the 1 N insert or the 4R repeat increases aggregation in vitro (Zhong et al. 2012).

These structural variations also affect susceptibility to post-translational modifications (PTMs). Inclusion of exons 2, 3, and 10 alters tau’s conformation, adding modifiable residues that influence microtubule affinity and aggregation propensity (Alquezar et al. 2021; Alhadidy et al. 2025). A balanced expression of 3R and 4R isoforms is essential for cellular homeostasis (Miguel et al. 2019; He et al. 2020), but the specific contributions of each isoform to tauopathy pathogenesis remain poorly defined.

Functionally, the microtubule-binding and C-terminal regions are highly conserved across species (Trushina et al. 2019), underscoring tau’s central role in microtubule regulation, tubulin polymerization, and cytoskeletal stability (Murphy et al. 1977). Tau also contributes to axonal transport, neuronal morphology, and neurogenesis. In contrast, the N-terminal and proline-rich regions are more variable and mediate interactions with a broad range of cellular partners (Trushina et al. 2019). Proteomic studies have identified over a hundred tau-interacting proteins associated with apoptosis, dendritic development, transcription, and proteasomal regulation (Sinsky et al. 2020; Shapley et al. 2023; Younas et al. 2024). The N-terminal domain also governs association with the axonal plasma membrane (Brandt et al. 1995; Maas et al. 2000), a process influenced by hyperphosphorylation, pathogenic mutations, and β-amyloid aggregates (Eidenmüller et al. 2001; Weissmann et al. 2009; Gauthier-Kemper et al. 2011).

Biophysically, tau is a soluble, intrinsically disordered protein with a dipolar charge distribution, negative at the N-terminus and positive in the proline-rich, microtubule-binding, and C-terminal regions (Kanaan et al. 2020; Abasi et al. 2024). This flexible structure makes it prone to misfolding and aggregation under poorly understood conditions (Silva and Haggarty 2020; Zheng et al. 2024). Tau aggregates —hallmarks of tauopathies— accumulate in neurons, glial cells, or the extracellular space (Chung et al. 2021).

Tau undergoes various post-translational modifications (PTMs) that regulate its function, localization, interactions, and aggregation (Ye et al. 2022; Kyalu Ngoi Zola et al., 2023). Abnormal increases in PTMs can disrupt tau’s interaction with microtubules and promote its aggregation (Ercan-Herbst et al. 2019; Alquezar et al. 2021). Among these modifications, phosphorylation is the most extensively studied. Tau phosphorylation status is determined by the activity of kinases such as glycogen synthase kinase 3 beta (GSK3β), CDK5, and PKA, and is developmentally regulated. During fetal development, tau is highly phosphorylated without functional impairment or increased aggregation. As brain development progresses, Tau phosphorylation levels decrease and remain at basal levels due to the high activity of phosphatases such as PP2A, PP2B, and PP5 (Yu et al. 2009; Su et al. 2024).

The effects of tau phosphorylation are highly context-dependent, reflecting a dual and sometimes opposing role in neuronal function and pathology. Several studies have reported seemingly contradictory effects of tau phosphorylation. Some evidence indicates that phosphorylation drives aggregation and mislocalization, contributing to neurodegeneration, whereas other reports show that selective phosphorylation can stabilize tau and support normal neuronal function.

On one hand, phosphorylation at specific residues, such as Ser202/Thr205, Thr231, and Ser422, can induce conformational changes that expose hydrophobic regions in the microtubule-binding domain, promoting intermolecular interactions, nucleation, and fibrillization, while decreasing tau’s affinity for microtubules and shifting it toward a soluble, aggregation-prone state (Pooler et al. 2012; Ulrich et al. 2018; Soto-Faguás et al. 2021; Man et al. 2023; Abasi et al. 2024). These modifications also disrupt axonal targeting signals, leading to mislocalization of tau in dendrites, synaptic terminals, and nuclei, where it can interfere with chromatin organization, transcriptional regulation, and membrane interactions, thereby contributing to synaptic dysfunction and neuronal vulnerability (Gauthier-Kemper et al. 2011; Pooler et al. 2012; Ulrich et al. 2018; Liu et al. 2020; Soto-Faguás et al. 2021).

On the other hand, site-selective and developmentally regulated phosphorylation can be protective: during fetal development, tau is physiologically hyperphosphorylated at multiple sites, maintaining its soluble, dynamic state and preventing pathological self-association through increased electrostatic repulsión (Yu et al. 2009; Su et al. 2024). Moreover, phosphorylation can modulate tau’s binding kinetics to microtubules, facilitating rapid detachment and reattachment that support axonal transport and cytoskeletal remodeling, while potentially shielding Tau from other aggregation-promoting modifications such as acetylation or truncation (Su et al. 2024).

This duality underscores that the functional consequences of Tau phosphorylation depend critically on the specific residues modified, the developmental or pathological context, and the combination of post-translational modifications, highlighting the importance of analyzing site-specific phosphorylation patterns rather than overall phosphorylation levels to understand tau biology and its role in neurodegenerative disease.

Phosphorylation occurs sequentially at specific residues—for example, Ser404 and Ser400 must be modified before Ser396 (Li and Paudel 2006)—and patterns vary with disease progression, with residues Ser199, Ser202/Thr205, Thr231, and Ser422 showing strong correlation with Braak stage III (Neddens et al. 2018). Understanding tau phosphorylation thus requires considering both site-specific and combinatorial modification patterns under physiological and pathological conditions.

Beyond phosphorylation, tau undergoes additional PTMs—including isomerization, glycosylation, nitration, acetylation, oxidation, polyamination, sumoylation, cleavage, and ubiquitination (Alquezar et al. 2021; García-Cruz et al. 2025)—which shape its structure, localization, and function (Alquezar et al. 2021; Buchholz and Zempel 2024). Although the precise biological relevance of many of these modifications remains to be elucidated, they are clearly part of a complex regulatory network influencing Tau’s role in health and disease.

### Tau in Mitochondria

Tau has been considered predominantly localized in the axons of neurons under physiological conditions (Iwata et al. 2019). However, accumulating evidence has revealed its presence in other cellular compartments, including the nucleus, mitochondria, synapses, and somatodendritic regions (Papasozomenos and Binder 1987; Cieri et al. 2018; Abasi et al. 2024). The abnormal redistribution of tau outside the axonal compartment is believed to be a distinctive feature associated with tauopathies such as Alzheimer´s disease (AD), Progressive Supranuclear Palsy (PSP) and Pick’s disease (Martínez-Maldonado et al. 2021; Kawles et al. 2024). Given tau’s structural versatility, it is possible that it performs as yet undescribed cellular functions in these various subcellular locations. The broad distribution of tau protein in the cell suggest that it serve in a variety of physiological and pathological functions. Notably, the link between tau and mitochondria has garnered increasing interest, as mitochondrial dysfunction is recognized as an early hallmark of neurodegenerative diseases.

An effective strategy for elucidating the functions of tau involves mapping its interactome, as proteins typically operate within coordinated networks of molecular interactions (Nada et al. 2024). This approach has proven valuable in identifying biological processes involving tau and elucidating its role in the pathology of tauopathies (Sinsky et al. 2021). While tau is best known for its role in stabilizing microtubules through binding to tubulin (Kohl et al. 2024), it also interacts with a wide variety of proteins like Fyn, ApoE3, VDAC1, Drp1, Opa1, Tomm40 and PLCγ (Tang et al. 2020; Tracy et al. 2022; Perez-Corredor et al. 2025), suggesting its participation in numerous signaling pathways. As a result, tau has been increasingly recognized as a scaffold protein (Mueller et al. 2021).

Although relatively few studies have comprehensively characterized the full network of tau interactions, several have consistently reported its association with mitochondrial proteins such as ANT1, ATP5A and cytochrome c (Liu et al. 2016; Tracy et al. 2022; Younas et al. 2024). These interactions impair mitochondrial bioenergetics, disrupting ATP production and its cytosolic availability (Torres et al. 2021), events that are characteristic of Alzheimer’s pathology (Venkataraman et al. 2022). Immunodetection studies have confirmed the presence of tau in mitochondrial fractions (Cieri et al. 2018; Trease et al. 2022), providing evidence of its physical localization within these organelles, where it appears to participate in complex protein-protein interactions. Notably, all major forms of tau, including native, truncated, misfolded, and mutated variants, have been detected in mitochondria (Amadoro et al. 2014; Torres et al. 2021; Tracy et al. 2022).

Although the mechanism of tau import into mitochondria and its precise localization within mitochondrial subcompartments remain under debate, several studies have reported its association with the outer mitochondrial membrane, the intermembrane space, and the mitochondrial matrix (Cieri et al. 2018; Torres et al. 2021; Trease et al. 2022; Castillo-Casaña et al. 2025). Tau has been shown to interact directly with TOMM40 and TIMM23, the outer and inner mitochondrial membrane translocases, respectively, without impairing protein import or respiratory function (Tracy et al. 2022; Needs et al. 2023). This interaction suggests the possible involvement of Tom40 and Tim23 in the mitochondrial tau import pathway. More recently, our group demonstrated in yeast that the cytoplasmic chaperones Ssa1 and Ydj1, together with the disaggregase Hsp104, play a central role in the mitochondrial import of tau (Castillo-Casaña et al. 2025). Nevertheless, several fundamental questions remain unanswered, including how tau translocates into mitochondria, how post-translational modifications influence its mitochondrial targeting and interactions, and whether different tau isoforms localize to specific mitochondrial subcompartments.

### Tau Involvement in Mitochondrial Transport

**Fig. 1:**
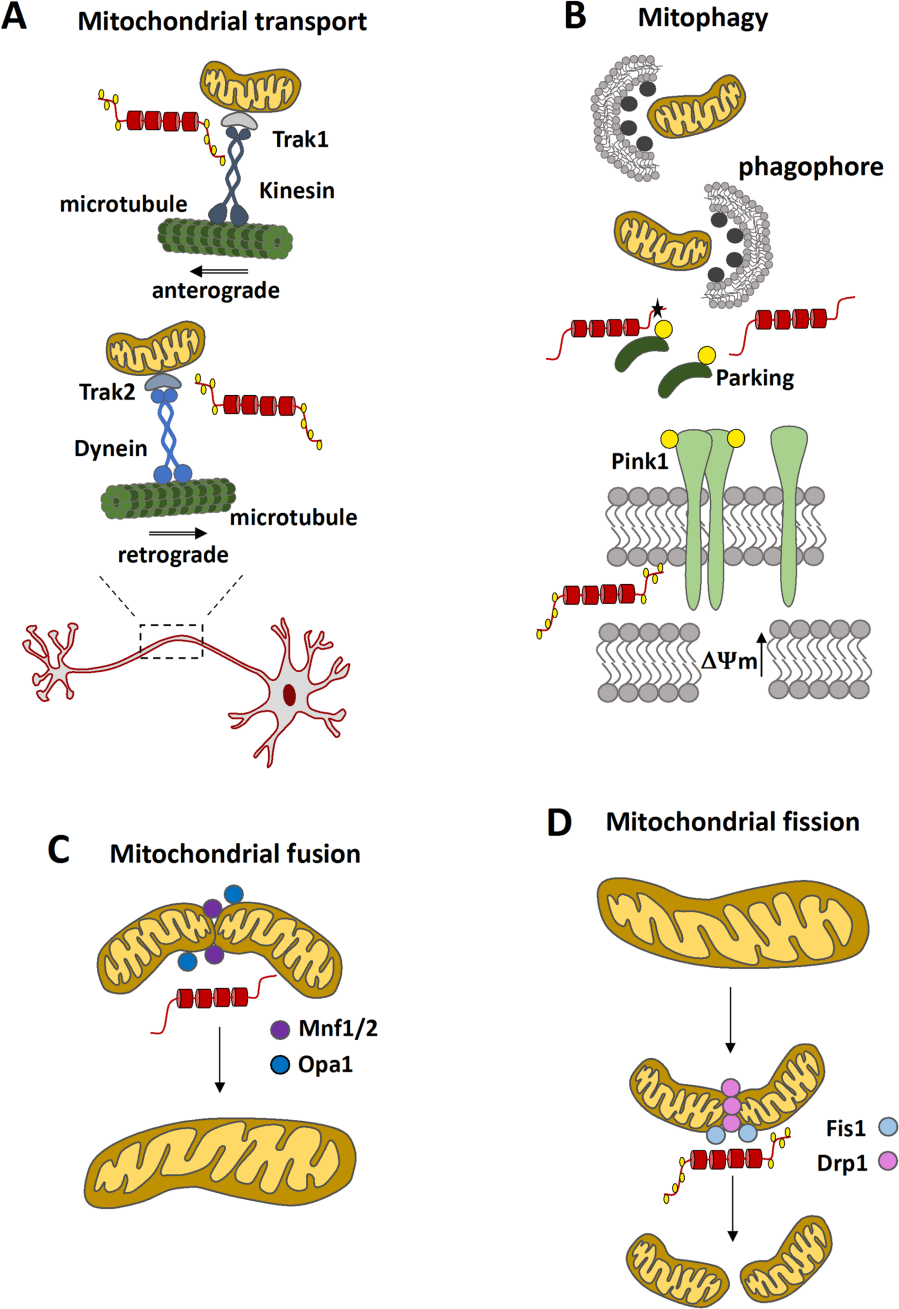
Effects of Tau protein on mitochondrial dynamics and clearance. Tau protein disrupts key mitochondrial processes, including transport, dynamics, and clearance. **A**. Tau affects mitochondrial transport by interfering with motor proteins responsible for anterograde and retrograde movement along microtubules, thereby impairing mitochondrial trafficking in axons. **B**. Tau impairs mitochondrial clearance by disrupting mitophagy, particularly through interactions with components of the PINK1-Parkin pathway. **C**. Tau influences mitochondrial fusion by interacting with Opa1 and Mnf1/2. **D**. Tau inhibits mitochondrial fission by interacting with Drp1. Tau protein is depicted in red, phosphate groups are shown as yellow circles, and mutated tau is indicated with a black star

Tau plays a critical role in regulating the interaction and movement of motor proteins such as kinesins and dyneins along microtubules, thereby influencing the transport of organelles, including mitochondria, to various cellular locations (Beaudet et al. 2024). In neurons, mitochondrial transport occurs in two primary directions: anterograde transport, from the soma to synaptic terminals, mediated by kinesin 1, and retrograde transport, from the terminals back to the soma, mediated by dynein (Reiss et al. 2024) (Fig. 1).

Evidence from AD models indicates that overexpression and accumulation of human tau disrupt anterograde mitochondrial transport and alter mitochondrial distribution within the cell (Kanaan et al. 2011; Kopeikina et al. 2011). This dysfunction is linked to increased activity of GSK3β, which not only induces tau hyperphosphorylation (Alquezar et al. 2021) but also promotes mitochondrial stationary behavior without affecting their intrinsic velocity (Morel et al. 2010; Kanaan et al. 2011). Notably, GSK3β is found to be overactive in the brains of Alzheimer’s patients (Zhou et al. 2022), which may be a factor contributing to microtubule destabilization and further impairment of mitochondrial transport. In fact, GSK3β-mediated tau phosphorylation at the three residues, Ser199/Ser202/Thr205, increases the spacing between microtubules, contributing to decrease mitochondrial mobility (Shahpasand et al. 2012). Similarly, phosphomimetic tau mutants, and pseudo-hyperphosphorylated tau forms, negatively affect mitochondrial transport in human circadian clock neurons and embryonic stem cells (Mertens et al. 2013; Zhang et al. 2022).

In addition to phosphorylation, tau mutations and truncations also impair mitochondrial dynamics. Fronto-temporal associated tau mutations such as P301L, R406W, N279K and K3691 have been shown to reduce anterograde mitochondrial transport in stem cells (Iovino et al. 2015; Ittner et al. 2008; Nakamura et al. 2019). On the other hand, the expression of GFP-T4C3—a truncated tau construct—impairs both retrograde and anterograde mitochondrial movement. This disruption correlates with decreased levels of TRAK2, a mitochondrial adaptor protein, increased mitochondrial association of TRAK2, and reduced ATP production (Quintanilla et al. 2020). Collectively, these findings suggest that biochemical modifications of tau, whether due to mutations, aberrant post-translational modifications, or proteolytic cleavage, significantly impair mitochondrial transport in both directions. Although the precise mechanisms remain incompletely understood, it is likely that toxic tau variants contribute to microtubule destabilization and interfere with accessory proteins critical for the coordination of mitochondrial-motor protein interactions. Given that mitochondrial movement is crucial for maintaining local ATP and calcium levels, particularly at synaptic terminals, impaired transport has been linked to axonal and dendritic dysfunction, reduced synaptic transmission and plasticity, excitotoxicity, and ultimately neuronal death (Silva et al. 2021).

### The Impact of Tau on Mitochondrial Morphology

Mitochondria are highly dynamic organelles capable of altering both their internal architecture and overall network morphology in response to physiological demands (Lyra-Leite et al., 2021). These structural adaptations enable mitochondria to optimize functions such as ATP production and metabolite synthesis based on cellular needs (Golombek et al. 2024). Notably, mitochondrial plasticity is cell-type dependent: neurons, for example, prioritize efficient ATP generation, whereas liver cells emphasize biosynthetic pathways for macromolecule production. Although the molecular mechanisms governing mitochondrial spatial organization remain incompletely understood, structural modulation occurs at multiple levels—including changes in cristae morphology, matrix and nucleoid volume, and overall organelle dynamics. Under pathological conditions, these features are often compromised, leading to impaired mitochondrial function and disrupted interactions with neighboring organelles (Golombek et al. 2024). Alterations such as mitochondrial elongation and volume reduction are commonly observed in neurodegenerative diseases such as Alzheimer’s, as well as during aging (Jenkins et al. 2024). Increased volume and surface area of mitochondria along with disrupted outer membrane integrity, have been reported in the hippocampus from aged mice (Torres et al. 2021). These morphological abnormalities were associated with a significant accumulation of phosphorylated tau, implicating tau in mitochondrial structural dysfunction. However, it remains to be determined whether these effects result from direct interactions between tau and mitochondria or are secondary to broader cellular stress.

Similarly, the neurotoxic tau fragment NH2-26–230 (Amadoro et al. 2014) induces pronounced structural alterations when expressed in neuroblastoma cells. This fragment accumulates in synaptosomal mitochondria, which exhibit a pale, rounded morphology, reduced size, and extensive internal disorganization. Affected mitochondria display fragmented and irregular matrices, disrupted or absent cristae, interrupted intermembrane spaces, and fractured outer membrane (Amadoro et al. 2014). Although the precise mechanisms through which pathological tau species induce mitochondrial damage remain unclear, accumulating evidence supports a strong association between tau mitochondrial localization and both structural and functional mitochondrial impairment.

### Tau in Mitochondrial Dynamics

The influence that tau exerts on mitochondrial structure (Torres et al. 2021), transport, and distribution (Kanaan et al. 2011; Kopeikina et al. 2011), alongside its interactions with mitochondrial proteins (Tracy et al. 2022), suggests a potential role in regulating mitochondrial dynamics (Fig. 1). One key player in mitochondrial dynamics is the GTPase Drp1, which mediates mitochondrial fission. Notably, a direct interaction between Drp1 and tau phosphorylated at Ser396 has been reported in post-mortem brain from AD patients and in 13-month-old 3xTg-AD mice. This interaction is associated with increased Drp1 activity and excessive mitochondrial fragmentation (Manczak and Reddy 2012a; Espino de la Fuente-Muñoz et al., 2020). Tau has also been shown to co-immunoprecipitate with the mitochondrial fusion-related proteins OPA1 (optic atrophy 1) and Mitofusin 1 (Mfn1), indicating a potential regulatory role in mitochondrial fusion (Hu et al. 2016). Furthermore, the expression and mitochondrial accumulation of the neurotoxic tau fragment NH2-26-230 results in a significant reduction in the levels of key fusion proteins, including OPA1, Mfn1, and Mfn2, as detected by immunodetection assays (Amadoro et al. 2014) suggesting impaired mitochondrial fusion capacity. However, contradictory findings have also been reported; it has been observed that tau accumulation may promote mitochondrial fusion under certain conditions (Li et al. 2016). These discrepancies highlight the complexity of tau’s influence on mitochondrial dynamics and underscore the need for further studies to clarify the mechanisms involved.

Mitochondrial dynamics are essential for maintaining mitochondrial quality control, energy metabolism, and overall cellular homeostasis (Wang et al. 2023). In neurons, excessive mitochondrial fragmentation not only disrupts ATP production and promotes reactive oxygen species (ROS) generation but also contributes to mitochondrial DNA loss and retrograde translocation of mitochondria to the soma. These events are linked to neuroinflammation and neurodegeneration (Wang et al. 2020). Therefore, tau may act as a critical regulator of mitochondrial dynamics, with implications for both mitochondrial metabolism and neuronal health. Understanding the molecular mechanisms by which tau influences mitochondrial fusion and fission could pave the way for novel therapeutic strategies aimed at restoring mitochondrial homeostasis and mitigating neurodegeneration in tauopathies.

### Tau in ATP Production and Translocation

**Fig. 2:**
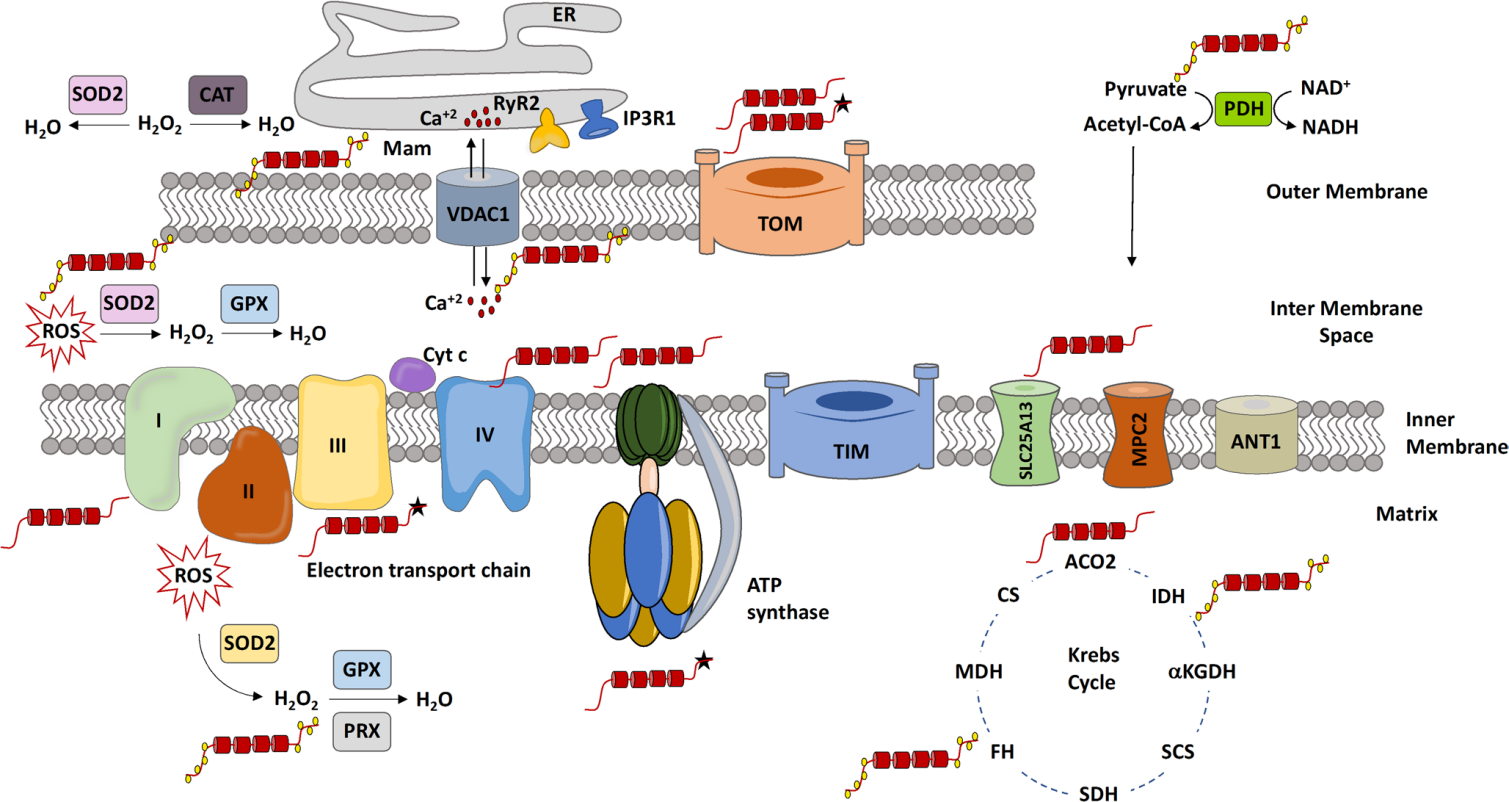
Effects of tau protein on mitochondrial bioenergetics. Tau protein disrupts mitochondrial energy homeostasis through multiple mechanisms. It alters the mitochondrial energy balance by interacting with components of the electron transport chain and oxidative phosphorylation system. Tau also interferes with the transport of metabolic substrates and impairs the Krebs cycle. In addition, tau interacts with mitochondrial import complexes, affecting the translocation of nuclear-encoded proteins into mitochondria. It disrupts the physical and functional interaction between the mitochondrial outer membrane and the endoplasmic reticulum, thereby altering intracellular Ca²⁺ homeostasis. Furthermore, tau promotes the production of reactive oxygen species (ROS) and impairs the activity of key antioxidant enzymes, contributing to oxidative stress. Mam: Mitochondria-associated membranes. Tau protein is depicted in red, phosphate groups are shown as yellow circles, and mutated tau is indicated with a black star

A primary function of mitochondria is the generation of ATP through oxidative phosphorylation, though these organelles also participate in numerous physiological processes essential for maintaining cellular homeostasis and survival (Valenti and Atlante 2022). The expression of the six human tau isoforms in Htau transgenic mice significantly impairs the maximal respiratory capacity of synaptic mitochondria, without altering the total mitochondrial content (Trease et al. 2022) (Fig. 2). This suggests that tau exerts a detrimental effect on mitochondrial bioenergetics, potentially through its interaction with several components of the electron transport chain (ETC), including complexes I, III, and IV, ATP synthase (complex V), and cytochrome c (Tracy et al. 2022). Consistent with these findings, it was reported that mitochondrial accumulation of tau phosphorylated at Ser396/Ser404 in 18-month-old mice coincides with reduced levels of mitochondrial complexes I and IV, along with an increase in complex V (Torres et al. 2021). Similarly, the expression of the neurotoxic tau fragment NH2-26–230 leads to decreased levels of cytochrome C and its associated complex IV subunit COX IV (Amadoro et al. 2014). Perturbations in the stoichiometry of ETC complexes can impair the formation and stability of respirasomes, thereby reducing ATP synthesis and increasing electron leakage and reactive oxygen species (ROS) production (Bochkova et al. 2025). In fact, both the phosphorylated form of tau, PHF-1 and the NH2-26–230 fragment of tau have been shown to decrease mitochondrial membrane potential and ATP production while increasing ROS levels in vitro and in vivo (Fig. 2) (Amadoro et al. 2014; Torres et al. 2021). Nonetheless, contradictory findings have been reported; for example, tau overexpression was associated with increased mitochondrial membrane potential in a different study (Hu et al. 2016), indicating that the effects of tau on mitochondrial function may be context dependent.

The tricarboxylic acid (TCA) cycle, which generates reducing equivalents (NADH and FADH₂) via the oxidation of acetyl-CoA, is another fundamental process in mitochondrial energy metabolism. These reducing equivalents feed into the ETC to generate the proton gradient required for ATP production (Arnold and Finley 2023). Both native and phosphorylated forms of tau have been shown to interact with several key enzymes of the TCA cycle, including pyruvate dehydrogenase (PDH), citrate synthase (CS), aconitase (ACO2), fumarate hydratase (FH), succinate dehydrogenase (SDH), isocitrate dehydrogenase (IDH), and malate dehydrogenase (MDH) (Fig. 2) (Liu et al. 2016; Drummond et al. 2020; Tracy et al. 2022). Although the precise biological implications of these interactions remain unclear, transcriptomic analyses of brain and peripheral blood cells from AD patients have shown a marked downregulation of genes encoding TCA cycle enzymes. In AD mouse models, the expression of IDH, PDH, MDH, 2-oxoglutarate dehydrogenase, and succinyl-CoA synthetase was strongly negatively correlated with tau pathology (Jia et al. 2023). Consistently, in hTau transgenic mice expressing the P301S mutation, there is a reduced expression of oxidative phosphorylation and TCA cycle enzymes, further implicating tau in the functional deterioration of these metabolic processes (Tsumagari et al. 2022). Conversely, the accumulation of certain TCA intermediates such as succinate and fumarate can activate pro-tumorigenic signaling cascades. Thus, any disruption in the enzymatic function of TCA components can profoundly impact mitochondrial performance, cellular homeostasis, and cell fate.

In addition to its influence on the ETC and TCA cycle, tau has been shown to interact with adenine nucleotide translocase (ANT), a key protein in the inner mitochondrial membrane responsible for exchanging cytosolic ADP for mitochondrial ATP (Fig. 2) (Tracy et al. 2022). Functional disruption of ANT impairs ATP export to the cytoplasm, precipitating bioenergetic failure, particularly in high-energy-demanding cells such as neurons (Chen et al. 2023). The activity of ANT is severely impaired by the tau-derived peptide NH2-26–44, as demonstrated in primary cerebellar granule cell cultures (Atlante et al. 2008) and postmortem AD brain tissue (Amadoro et al. 2012). This tau fragment co-immunoprecipitates with ANT-1 and can form a complex with β-amyloid and cyclophilin D. Interestingly, it binds to a non-catalytic site on ANT, causing conformational changes that inhibit its function (Atlante et al. 2010). Mechanistically, NH2-26–44 appears to act as a competitive inhibitor of ANT-1 (Amadoro et al. 2012). Notably, this regulatory effect occurs during apoptosis, where caspase-mediated cleavage of tau generates the NH2-26–44 fragment, thereby impairing ANT function (Atlante et al. 2010). Altogether, these findings underscore that both native tau and its pathological variants can interfere with mitochondrial bioenergetics by modulating key proteins involved in ATP production and transport, including components of the TCA cycle, ETC, oxidative phosphorylation machinery, and transmembrane ADP/ATP exchangers such as ANT. However, the molecular mechanisms underlying tau-mediated regulation of these processes remain incompletely understood.

### Tau Effects on Mitochondria-Nucleus Signaling and Mitophagy

A healthy mitochondrial network is maintained through mechanisms that repair or eliminate damaged mitochondria. Two key quality control pathways involved in this process are the mitochondrial unfolded protein response (UPR^mt^) and mitophagy. The UPR^mt^ is a mitochondria-to-nucleus signaling pathway that triggers transcriptional and metabolic adaptations in response to mitochondrial proteotoxic stress (Suárez-Rivero et al. 2022). Activation of this stress response has been implicated in the pathogenesis of several neurodegenerative diseases, including Parkinson’s, Huntington’s, and AD (Patergnani et al. 2022). Surprisingly, there is limited evidence directly linking tau to the UPR^mt^. However, treatment with ISRIB (an integrated stress response inhibitor) in PITRM1^−/−^ brain organoids lead to the accumulation of phosphorylated tau, suggesting a potential connection between tau and UPR^mt^ signaling (Pérez et al. 2021). Additionally, our research group has shown that accumulation of the 0N3R-Tau isoform in the mitochondrial compartments of *Saccharomyces cerevisiae* activates the mitochondrial retrograde response (Castillo-Casaña et al. 2025). The interplay between tau and the UPR^mt^ remains a largely unexplored area and warrants further investigation.

In mammals, the collapse of the mitochondrial membrane potential (ΔΨm) not only impairs oxidative phosphorylation (Rieger et al. 2021) but also triggers a selective degradation process known as mitophagy (Matsuda et al. 2010) (Fig. 1). Under physiological conditions, PINK1 (PTEN-induced kinase 1) is continuously imported into healthy mitochondria and degraded by mitochondrial proteases. However, in depolarized or damaged mitochondria, this import and degradation are inhibited, leading to the accumulation of PINK1 on the outer mitochondrial membrane (Matsuda et al. 2010). This accumulation promotes the recruitment of the E3 ubiquitin ligase PARKIN, which ubiquitinates outer membrane proteins, marking the dysfunctional mitochondria for clearance by the autophagic machinery (Basak and Holzbaur 2025). Notably, overexpression and mitochondrial accumulation of tau in human embryonic kidney cells has been shown to increase mitochondrial membrane potential, thereby preventing PINK1 stabilization and PARKIN recruitment. As a result, mitophagy is impaired under these conditions (Hu et al. 2016). Similarly, both wild-type tau (WT-Tau) and the mutant P301L-Tau have been reported to interact with PARKIN, sequestering this E3 ubiquitin ligase in the cytosol and thereby inhibiting mitophagy (Fig. 1) (Cummins et al. 2019). Expression of phosphomimetic tau variants, such as 2EC (S396E/S404E) and 2EM (T231E/S235E), also impairs oxidative stress-induced mitophagy by interfering with the activity of the mitophagy receptor FKBP8 (Isei et al. 2024). Comparable inhibitory effects on mitophagy have been observed in *C. elegans* expressing tau mutants that mimic post-translational modifications, including T231E and K274/281Q (Guha et al. 2020). In contrast, neurons expressing the neurotoxic tau fragment NH2-26–230 show enhanced mitophagic flux (Amadoro et al. 2014), suggesting that tau influences on mitophagy may be variant-specific and context-dependent. These findings underscore the complexity of tau-mediated regulation of mitochondrial quality control mechanisms and highlight the need for further investigation into the differential roles of tau species in mitophagy.

### Tau in Mitochondrial Ca^2+^ Regulation

In mammals, the maintenance of mitochondrial Ca²⁺ homeostasis is a tightly regulated process that relies on the coordinated activity of various ion channels and exchangers. One key component is the voltage-dependent anion channel (VDAC), located on the outer mitochondrial membrane, which facilitates the transfer of Ca²⁺ and other metabolites (Romero-Garcia and Prado-Garcia 2019). Additionally, mitochondrial Ca²⁺ uptake is influenced by inter-organelle communication, particularly through structures known as mitochondria-associated membranes (MAMs), which form functional contact sites between the mitochondria and the endoplasmic reticulum (ER) and enable efficient Ca²⁺ transfer between these organelles (Fig. 2) (Vrijsen et al. 2022).

MAMs are dynamic structures that, beyond facilitating Ca²⁺ transfer and signaling, also regulate cellular processes including lipid metabolism, apoptosis, mitochondrial dynamics, ROS production, and the endoplasmic reticulum unfolded protein response (UPR^ER^) (Fernandes et al. 2023). Dysregulation of calcium signaling, and structural or functional disruption of MAMs have been implicated in the pathogenesis of AD. Notably, proteins such as presenilins, amyloid precursor protein (APP), and the Aβ peptide have been shown to perturb calcium homeostasis through interactions with key MAM components, including SERCA (sarco/endoplasmic reticulum Ca²⁺-ATPase) and IP₃R (inositol 1,4,5-trisphosphate receptor) (Green et al. 2008; Cheung et al. 2010; Fernandes et al. 2021). In particular, injection of synaptotoxic amyloid beta oligomers into hippocampal neuron cultures induces the accumulation of the RyR2 (type-2 ryanodine receptor) calcium channel, IP_3_R1 and VDAC in the MAMs (More et al. 2018). These changes lead to a deregulation of intracellular calcium levels, which in turn enhance AD´s hallmarks such as tau phosphorylation and Aβ peptide accumulation, thereby exacerbating neurotoxicity (Querfurth and Selkoe 1994; Pierrot et al. 2006).

The relationship between tau, mitochondria-associated membranes (MAMs), and mitochondrial Ca²⁺ homeostasis is an interesting topic for research. For example, tau interactome studies have identified direct interactions between tau and several key MAM components, including VDAC1 and mitofusins 1 and 2 (Hu et al. 2016; Drummond et al. 2020). VDAC1 levels are known to increase with age in the brains of AD patients and in various AD mouse models. Notably, tau phosphorylated at Ser202/Thr205 has been shown to bind to and inhibit VDAC1, thereby contributing to mitochondrial dysfunction (Fig. 2) (Manczak and Reddy 2012b). Importantly, partial genetic reduction of VDAC1 in VDAC1⁺/⁻/Tau(P301L) mice significantly improves synaptic function, enhances autophagy and mitophagy markers, and restores cognitive and motor performance when compared to Tau(P301L) mice alone (Vijayan et al. 2022). These findings highlight VDAC1 as a promising therapeutic target for the treatment of tauopathies (Varughese et al. 2021).

Recent studies have revealed that tau interacts with the modulator SAH hydrolase–like protein 1/IP_3_R–binding protein (AHCYL1/IRBIT), a regulator of IP₃R function. This interaction alters the spatial proximity between tau and IP₃R, potentially compromising IP₃R activity and the structural integrity of MAMs (Wischhof et al. 2022). Although the precise impact of this interaction on calcium signaling and MAM function remains to be fully elucidated, current evidence suggests a potential negative regulatory effect. Additionally, a truncated form of tau (2N4RΔC20) has been localized to the outer mitochondrial membrane and the intermembrane space, where it has been shown to disrupt endoplasmic reticulum (ER) calcium homeostasis and impair ER–mitochondria contacts (Cieri et al. 2018). These findings support the emerging view that pathological tau species may interfere with MAM integrity and ER–mitochondria communication, further contributing to mitochondrial dysfunction in tauopathies.

The expression of the P301L tau mutant has been shown to impact ER–mitochondria communication in a context-dependent manner. In Pr5 mice and human SH-SY5Y neuroblastoma cells, P301L expression reduces the number of ER–mitochondria contact sites by disrupting interactions between vesicle-associated membrane protein-associated protein B (VAPB) and protein tyrosine phosphatase-interacting protein 51 (PTPIP51), key tethering components of mitochondria-associated membranes (MAMs) (Szabo et al. 2023). In contrast, studies in asymptomatic JNPL3 mice expressing the same tau variant suggest an increase in rough ER–mitochondria contacts (Perreault et al. 2009). Notably, no evidence of calcium homeostasis disruption was found in rTg4510 mice also expressing P301L, despite significant dendritic spine loss associated with tau pathology (Kopeikina et al. 2013). These seemingly contradictory findings may be due from differences in experimental models, disease stages, or methodologies, emphasizing the need for further investigation into tau’s role in ER–mitochondria crosstalk. Disruptions in these interorganelle interactions contribute to broader metabolic dysregulation and cellular stress, exacerbating neurodegenerative processes. Altogether, these findings highlight the complex and variant-specific effects of tau on mitochondrial calcium homeostasis and suggest that targeting tau-mediated MAM dysfunction could offer a promising neuroprotective strategy in AD and related tauopathies.

### Tau on ROS

Although mitochondria are a primary source of ROS during oxidative phosphorylation, particularly at complexes I and III, other cellular compartments also contribute to ROS production (Kozlov et al. 2024). At physiological levels, ROS function as essential signaling molecules involved in regulating gene expression, cell proliferation, and immune responses (Haque et al. 2019). However, due to their high reactivity, excessive ROS accumulation can damage lipids, proteins, and DNA, ultimately leading to cellular dysfunction and death (Kozlov et al. 2024). The mitochondrial accumulation of toxic tau species, including the PHF-1 and the NH2–26–230 fragment, has been shown to elevate both mitochondrial and cytosolic ROS, as well as increase of the GSSG/GSH ratio, a marker of oxidative stress (Fig. 2) (Amadoro et al. 2014; Torres et al. 2021).

Mitochondria counteract ROS through a robust antioxidant defense system including enzymes such as superoxide dismutase (SOD), catalase (CAT), glutathione peroxidase (GPx), and peroxiredoxins (Prx), to maintain redox balance (Kozlov et al. 2024). When this balance is disrupted, the resulting oxidative stress is a well-recognized contributor to neurodegenerative pathologies (Hong et al. 2024). Notably, tau has been found to interact with several antioxidant enzymes, both within neurofibrillary tangles and in mitochondrial compartments (Fig. 2) (Drummond et al. 2020; Tracy et al. 2022), which may help explain the reduced mitochondrial redox capacity observed in synaptosomes from the hippocampus and cerebral cortex of young and adult 3xTg-AD mice (Espino de la Fuente-Muñoz et al., 2020).

The relationship between ROS and tau pathology is complex and likely bidirectional: oxidative stress can modulate tau expression and post-translational modifications, while aberrant forms of tau exacerbate oxidative damage (Liu et al. 2015; Haque et al. 2019). The presence of tau within mitochondria, together with its interactions with both ROS-generating complexes and antioxidant systems, suggests a role in the pathophysiology of tauopathies through the disruption of redox homeostasis.

### Tau–mitochondrial Interactions and Implications for Therapeutic Strategies

Worldwide, more than 55.2 million people currently suffer from dementia, a number projected to reach 139 million by 2050, with AD accounting for 60–70% of these cases, making it an urgent global health challenge (WHO 2021). Current approaches focus on prevention, early diagnosis, and treatment, yet most patients are diagnosed at advanced and irreversible stages of the disease. The development of reliable biomarkers is therefore critical for pre-symptomatic detection, enabling timely patient care and management (Zhang et al. 2024). Present diagnostic tools rely on imaging, blood, and cerebrospinal fluid (CSF) biomarkers. β-amyloid peptide and phosphorylated tau deposition can be detected through magnetic resonance imaging and positron emission tomography, but these methods alone cannot reliably distinguish AD from other neurodegenerative diseases and require complementary clinical assessments (Odusami et al. 2024). CSF biomarkers offer an alternative for early diagnosis, while blood-based biomarkers are particularly attractive due to their accessibility, low cost, and minimally invasive nature. Examples include Aβ42, the Aβ42/Aβ40 ratio, total tau, and phosphorylated tau (P-tau181) (Zhang et al. 2024). Given the strong association between mitochondrial dysfunction and neurodegeneration, mitochondrial biomarkers such as cell-free mtDNA, neuroexosomal proteins (NDUFS3, SDHB), oxidative stress markers (SOD2), and mitochondrial dysfunction markers (e.g., COX, PMAIP1, FUNDC1, MAP1LC3A, CSNK2A1, VDAC1, CSNK2B, ATG5) have emerged as promising predictive tools, although their clinical utility still requires validation in large-scale trials (Wilkins et al. 2017; Chi et al. 2022; Ma et al. 2023; Ling et al. 2024; Di Lorenzo et al. 2025).

Treatment remains a cornerstone of AD management, yet no curative therapy currently exists. Approved drugs aim to alleviate symptoms and include cholinesterase inhibitors (Tacrine, Donepezil, Rivastigmine, Galantamine), NMDA receptor antagonists (Memantine), and anti-amyloid immunotherapies (Lecanemab, Donanemab) (Sims et al. 2023; Tang et al. 2023; Gao et al. 2024; Rabinovici et al. 2025). Notably, no drugs have been approved for tauopathies, though multiple investigational therapies are advancing in clinical trials. A comprehensive and detailed overview can be found in Harris et al. (2025). There is currently a reduced but evolving spectrum of therapeutic strategies targeting tau pathology, which can be grouped into several main categories; in this review, we highlight some selected representative examples of these approaches.

(A) Immunotherapies: These include monoclonal antibodies and vaccines that target extracellular tau or specific epitopes to block propagation or enhance clearance. High-affinity monoclonal antibodies against the paired helical filament (PHF-core) tau region (aa 297–391) inhibit pathological tau aggregation and seeding (Arastoo et al. 2024). Vaccine-based strategies include AADvac-1 (tau aa 294–305 conjugated to KLH), which was shown to be safe and immunogenic in a Phase I trial in mild to moderate AD, and ACI-35, a liposomal vaccine with a synthetic phospho-tau peptide (aa 393–408, pS396/pS404) that elicits strong phospho-specific antibody responses in animal models (Winblad et al. 2014). Additionally, antibody 43D (tau aa 6–18 epitope) has been shown to block p-tau seeding and spread in 3×Tg-AD mice (Dai et al., 2018).
(B) Tau aggregation inhibitors: These aim to prevent misfolding, oligomerization, or fibril elongation. Examples include LMTX (TRx0237), an oral methylthioninium derivative that reached phase 3 trials (Wischik et al. 2022); OLX-07010, which inhibits tau self-association and improves motor function in preclinical models (Davidowitz et al. 2025).
(C) Modulation of tau post-translational modifications (PTMs): This strategy targets hyperphosphorylation, acetylation, and other modifications. Kinase inhibitors such as ZDWX-25 (dual GSK-3β/DYRK1A inhibitor) and AS1842856 reduce tau phosphorylation (He et al. 2025; Liu et al. 2023). PP2A activators like sodium selenate enhance tau dephosphorylation (Cacabelos et al. 2024). HDAC6 inhibitors (e.g., MPT0G211 and CKD-504) promote tau acetylation and clearance (Fan et al. 2018; Choi et al. 2020), while inhibition of acetyltransferases (e.g., with salsalate or C646) mitigates pathological acetylation (Cacabelos et al. 2024).
(D) Reduction of tau expression or synthesis: RNA-targeting strategies, particularly antisense oligonucleotides (ASOs), aim to lower tau levels and reduce pathological substrate. BIIB080 (IONIS-MAPTRx / MAPTRx) targets MAPT mRNA via RNase H1 and achieved dose-dependent reductions in CSF total tau and p-tau181 in clinical trials, advancing to Phase 2 (CELIA), while NIO752 (Novartis) completed a Phase 1 trial in progressive supranuclear palsy (Harris et al. 2025).
(E) Enhancement of tau clearance and proteostasis: These strategies aim to activate degradation pathways such as autophagy, the proteasome, and chaperone systems. Tau-specific autophagic degraders like TA-3 reduce oligomeric tau and restore lysosomal function (Yoon et al. 2025). Modulation of the HSP70/Hsc70–CHIP system enhances ubiquitination and degradation of misfolded tau (Chesser et al. 2013), while macroautophagy inducers such as trehalose and rapamycin decrease insoluble tau and improve neuronal and behavioral outcomes.

Together, these complementary strategies define some current therapeutic landscape for tauopathies, reflecting both a diversification of mechanistic targets and the gradual clinical translation of disease-modifying interventions.

### Concluding Remarks

Under pathological conditions, tau protein plays a multifaceted and detrimental role in mitochondrial homeostasis. Its widespread impact on mitochondrial dynamics, bioenergetics, quality control, calcium regulation, and oxidative stress underscores its significant role in neurodegeneration. Mitochondrial function naturally declines with age creating a vulnerable environment in which pathological tau may exerts even greater toxicity, accelerating neuronal dysfunction. Future research should focus on elucidating the precise molecular mechanisms by which tau disrupts mitochondrial function and identifying therapeutic strategies to mitigate its toxic effects. Targeting tau interactions with mitochondrial components may offer promising avenues for preserving mitochondrial integrity, enhancing neuronal health, and developing effective treatments for tauopathies such as AD disease and related neurodegenerative disorders.

## Data Availability

No datasets were generated or analysed during the current study.
